# Experimental and Numerical Study of Nonlinear Lamb Waves of a Low-Frequency *S*_0_ Mode in Plates with Quadratic Nonlinearity

**DOI:** 10.3390/ma11112096

**Published:** 2018-10-25

**Authors:** Xiangyan Ding, Youxuan Zhao, Ning Hu, Yaolu Liu, Jun Zhang, Mingxi Deng

**Affiliations:** 1 College of Aerospace Engineering, Chongqing University, Chongqing 400044, China; dingxiangyan@cqu.edu.cn (X.D.); liuyaolu@cqu.edu.cn (Y.L.); mejzhang@cqu.edu.cn (J.Z.); dengmx65@yahoo.com (M.D.); 2 State Key Laboratory of Coal Mine Disaster Dynamics and Control, Chongqing University, Chongqing 400044, China

**Keywords:** ultrasonic nonlinearity, lamb waves, *S*_0_ mode, numerical simulation, experiment

## Abstract

This paper investigates the propagation of low-frequency *S*_0_ mode Lamb waves in plates with quadratic nonlinearity through numerical simulations and experimental measurements. Both numerical and experimental results manifest distinct ultrasonic nonlinear behavior which is mainly presented by the second harmonics. Meanwhile, we find that both the acoustic nonlinearity parameter and dispersion distance show the exponential decay trend with the increase of frequency-thickness. Moreover, the results reveal that the frequency is key to affect the acoustic nonlinearity parameter and dispersion distance with the same frequency-thickness. This study theoretically and experimentally reveals that nonlinear Lamb waves of the low-frequency *S*_0_ mode are feasible to quantitatively identify material weak nonlinearity in plates.

## 1. Introduction

The safety and durability of the key engineering structures, e.g., airplanes, pressure vessels, and high-speed trains, have been paid extensive attention to. Due to fatigue load, material degradation, the initiation and propagation of micro-cracks, micro-voids, etc. can often occur in those engineering structures in service life. Thus, there is an increasing demand to detect those small-scale defects using non-destructive testing methods. 

Because of the low sensitivity, conventional linear ultrasonic technology could be difficult to detect the above defects [1,2,3]. However, nonlinear ultrasonic technologies [4,5,6,7,8,9,10,11,12,13,14] are dramatically sensitive to material microstructures, which could be promising in overcoming this problem. Especially nonlinear Lamb-wave detection techniques [15,16,17,18,19,20,21,22,23,24,25,26,27,28,29,30,31,32,33,34,35,36,37,38,39], which can be employed for long-range monitoring and inspection in thin plate structures, have attracted extensive attention in the past two decades. 

Many efforts have been devoted to characterizing the micro-crack detection and early material degradation by higher harmonics of Lamb waves. Liu et al. [15] investigated experimentally the nonlinear acoustic effect on the crack depth. Shen et al. [16] numerically analyzed the process of Lamb waves interactions with fatigue cracks which can cause the nonlinear effect of higher harmonics and mode conversion. Deng et al. [17,18] and Matsuda et al. [19] declared that the matching phase velocities of fundamental and double frequency Lamb waves is necessary for an obvious second harmonic generation. Some researchers discussed the appropriate mode pairs for generation of the cumulative second harmonics in the circular tube damage/degradation quantitative assessment application [20,21]. Li et al. [22] found that the second harmonic generation of the circumferential guided wave is more sensitive to changes in the interfacial properties. Bermes et al. [23,24] measured second harmonic effects of Lamb waves in a metallic plate through a time-frequency representation with a hybrid wedge generation and laser interferometric detection system. In most studies, the *S*_1_ and *A*_1_ [25,26] modes are commonly used as the fundamental waves because the phase velocities of these two modes can be easily matched. Definitely, *S*_0_ and *A*_0_ modes [27,28,29,30] have great advantages due to the higher energy and longer-distance propagation. Castaings et al. [29] studied the interaction of the low order *A*_0_ and *S*_0_ with vertical cracks in aluminum plates. Hu et al. [30] investigated the nonlinear effects of low-frequency *S*_0_ Lamb wave in thin plates with randomly distributed micro-cracks through numerical simulations. Meanwhile, the nonlinear mixing wave method for Lamb waves has also been developed to evaluate material nonlinearity and micro-cracks. Jiao et al. [31] reported the application of nonlinear Lamb wave-mixing method to detect the micro-cracks in plates. Ishii et al. [32] have theoretically investigated the non-collinear interaction of plate wave modes when the nonlinear wave is propagating in elastic plates. Zhao et al. [33] applied one-way collinear mixing method to numerically investigate the propagation of Lamb waves in thin plates with quadratic nonlinearity.

The low-frequency Lamb waves could introduce higher energy and a longer propagating distance. However, the experimental studies on the *S*_0_ and *A*_0_ modes Lamb waves are still rarely reported. It is also a challenge to select a feasible frequency for characterizing material weak nonlinearity accurately and effectively by a low-frequency *S*_0_ mode Lamb wave. Therefore, in this work, we aim to numerically and experimentally investigate the propagation of the *S*_0_ mode Lamb wave at lower-frequency (200 kHz) in plates with quadratic nonlinearity. The relationship between dispersion distance and frequency-thickness is explored, and the influence of the frequency on the dispersion distance with the same frequency-thickness is further discussed.

## 2. Nonlinear Lamb Waves

**Figure 1:**
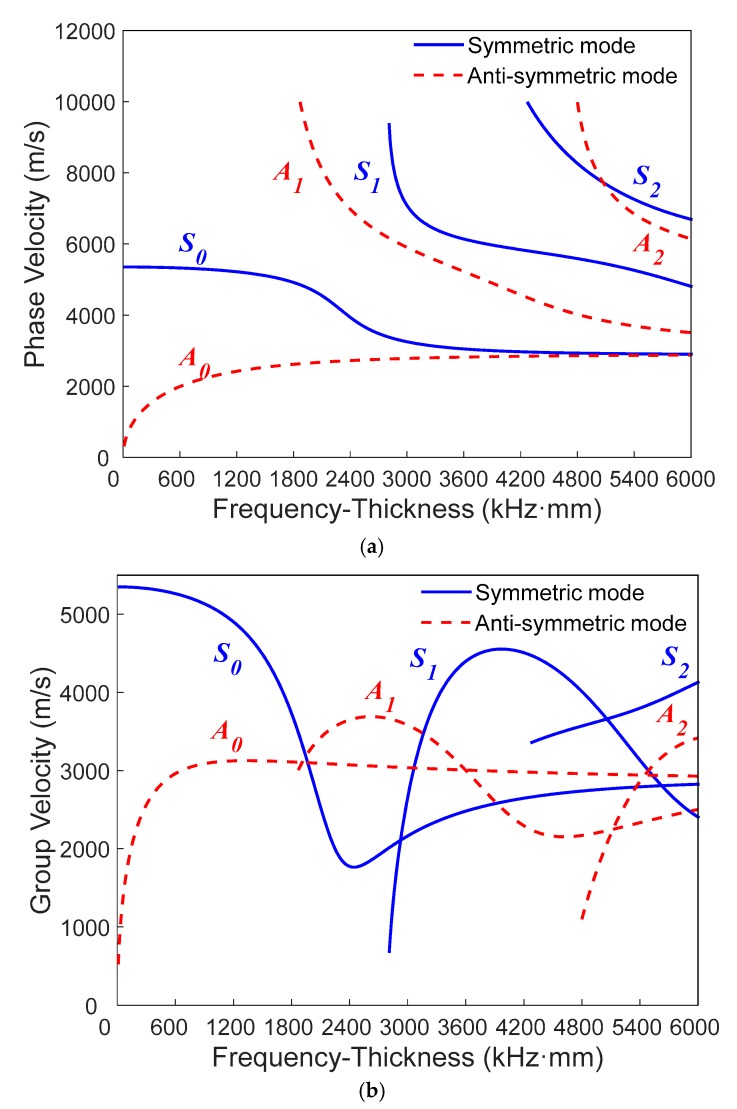
The dispersion curves of Lamb waves in an aluminum plate with a 2-mm thickness: (**a**) phase velocity; (**b**) group velocity.

The dispersion is a representative characteristic of Lamb waves which means that the velocity of Lamb wave depends on material properties and the frequencies of the waves. Figure 1 shows the dispersion curves of Lamb wave in an aluminum plate with a 2 mm thickness.

Lamb waves usually process various wave modes. However, it is crucial to select an appropriate mode with the characteristics of a long propagating distance, low energy attenuation, and stabilized mode in engineering applications. It is well-known that the *S*_0_ and *A*_0_ modes Lamb wave can carry more energy with a smaller energy attenuation. Figure 1 indicates that only the *S*_0_ and *A*_0_ modes exist in the low-frequency domain (0–1600 kHz⋅mm). Meanwhile, some studies have demonstrated that the phase velocity matching is one necessary condition to accumulate the second harmonic. Additionally, the velocity mismatch between the fundamental wave and second harmonic can lead to an asynchronous interaction, which is the reason for the sinusoidal behavior named as dispersion distance *L* [40]:

(1) $$L=\frac{2\pi }{\left|k_{d}\right|},k_{d}=k\left(2\omega \right)-2k\left(\omega \right)$$

where *k* is the wave number. When *k_d_* is small enough, the phase velocities of the fundamental wave and second harmonic are approximately equal. The resonance condition can be achieved and *L* can increase linearly to the critical value. Additionally, the linear accumulative distance commonly used in practice is 25% of the dispersion distance [28]. It is clear that the phase velocity of the *S*_0_ mode basically slowly changes in the low-frequency domain (0–800 kHz⋅mm) in Figure 1a. Thus, it is appropriate to choose the *S*_0_ mode in the frequency domain (0–800 kHz⋅mm) to evaluate the early material degradation based on second harmonic generation. In engineering applications, it is essential to validate the relationship between frequency-thickness and dispersion distance. However, the research on that is still rarely reported. Here, we investigate the potential of the *S*_0_ mode from the frequency range of 0–800 kHz⋅mm through numerical simulations and experimental measurements.

## 3. Numerical Simulation

The two-dimensional finite element model (FEM) is constructed to simulate the Lamb wave propagation in a plate with a quadratic material nonlinearity by the commercial FEM software ABAQUS (Version 6.14, Dassault Systèmes Simulia Corp., Providence, RI, USA).

**Figure 2:**
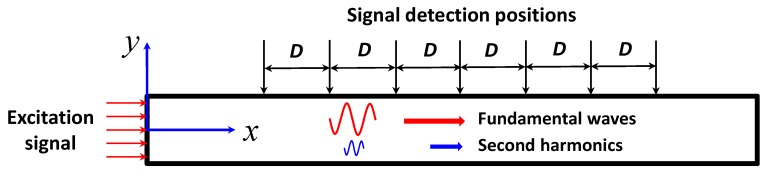
The schematic of Lamb waves propagation in a plate.

The problem of *S*_0_ mode Lamb waves propagating in a plate with a quadratic material nonlinearity is shown in Figure 2. An *S*_0_ mode wave pulse is generated by a dynamic displacement excitation on the left edge of the plate. The wave propagates along the *x* positive direction. Because of a quadratic nonlinearity, the second harmonic wave of the *S*_0_ mode is generated during the propagation of the fundamental *S*_0_ mode wave and finally received at different detection locations (the length *D* with a uniformly-spaced arrangement, *D* in this work is 25 mm).

The quadratic nonlinear elastic constitutive law with third-order constants is used [39], which is expressed using Voigt’s notation $C_{ijkl}=c_{IJ},\;C_{ijklmn}=c_{IJK}$:

(2) $$\sigma_{ij}=C_{ijkl}E_{kl}+\frac{1}{2}C_{ijklmn}E_{kl}E_{mn},$$

(3) $$C_{ijkl}=\lambda \delta_{ij}\delta_{kl}+\mu \left(\delta_{ik}\delta_{jl}+\delta_{il}\delta_{jk}\right),$$

(4) $$\begin{array}{c} C_{ijklmn}=\left(2l-2m+n\right)\delta_{ij}\delta_{kl}\delta_{mn}+\left(2m-n\right)\left(\delta_{ij}I_{klmn}+\delta_{kl}I_{mnij}+\delta_{mn}I_{ijkl}\right)\\ +\frac{n}{2}\left(\delta_{ik}I_{jlmn}+\delta_{il}I_{jkmn}+\delta_{jk}I_{ilmn}+\delta_{jl}I_{ikmn}\right). \end{array}$$

where *l*, *m*, and *n* are the Murnaghan third-order elastic constants and *E* is the Lagrangian or Green strain, I,J,K∈{1,2,3,4,5,6}, ij=11,22,33,23,31,12↔I=1,2,3,4,5,6.

The material properties of the aluminum (AL-6061-T6) plate used in the simulations are listed in Table 1. Considering the computational accuracy, each highest frequency wavelength requires at least 20 elements. The element size in simulations is set to *L*_max_ = 0.2 mm for the highest frequency 1200 kHz (the fundamental frequency is 600 kHz). The simulations with element size 0.15 mm and 0.1 mm are also investigated, which show the coincident accuracy but longer calculation time. Therefore, it is appropriate to select the element size as 0.2 mm for balancing the computational cost and accuracy. The rectangular region of 1000 mm × 2 mm is discretized by 50,000 four-node plane strain (CPE4R) elements in the FEM model.

ABAQUS/Explicit solver based on the central difference method is employed to solve the Lamb wave propagation in the time domain, which is conditionally stable. To ensure the accuracy of the solution, the stable time increment should be carefully chosen according to the time of the stress waves passing through the minimum element (3.9 × 10^−8^ s). Therefore, considering the efficiency and the accuracy, the stable time increment is set to $\Delta t=1.0\times 10^{-9}s$. Meanwhile, a double precision operation is also performed to reduce the accumulative error.

The left edge of the plate is applied a dynamic displacement excitation which is a tone-bust signal and can be expressed as $u\left(x,t\right)=A_{0}\sin\left(2\pi ft\right)\times \sin{\left(\pi ft/10\right)}^{2}$, where *A*_0_ is the amplitude of excitation signal (1 × 10^−4^ mm in this study [33,41]), and *f* is the frequency of excitation signal. The fundamental wave and second harmonic are collected at the detection positions. In addition, the distance between from the left edge to the right edge is large enough to maximally eliminate the influence of boundary reflection.

Moreover, the acoustic nonlinearity parameter $\beta =A_{2}/A_{1}^{2}$ [24] is used in this work, where *A*_1_ is the amplitude of the fundamental wave, and *A*_2_ is that of second harmonic.

Based on the concept mentioned in Section 2, we investigate the varying frequency-thickness cases with a constant 300 kHz and varying thickness (1.0–5.5 mm with the step of 0.5 mm). Additionally, cases of the same frequency-thickness (600 kHz·mm) with varying frequency are explored. More numerical case studies are not presented in this paper due to the same tendency.

## 4. Experimental Measurement

**Figure 3:**
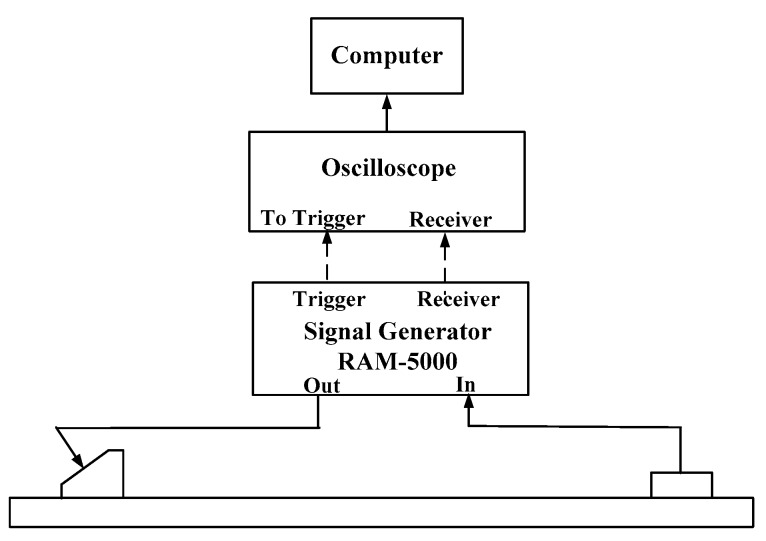
The schematic of the experimental setup.

In this section, a large number of experiments are employed to investigate the nonlinear behavior of Lamb waves. The schematic of the experimental setup is shown in Figure 3. A modular ultrasonic system RAM-5000 SNAP (RITEC Inc., Warwick, RI, USA) high power gated amplifier with two “RF burst” channels is used for the generation and detection of the nonlinear Lamb waves. 

**Figure 4:**
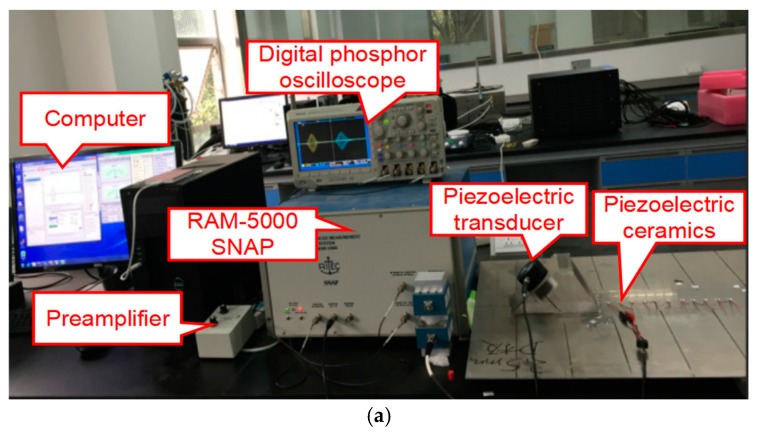

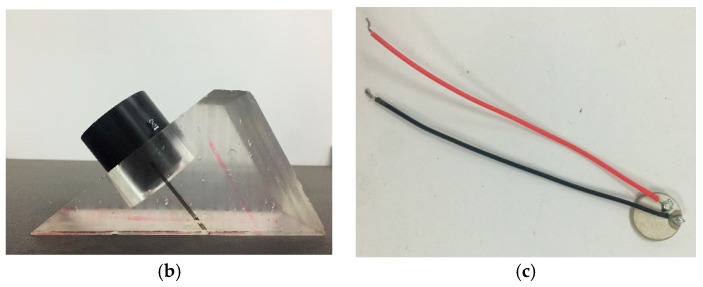
(**a**) The RAM-5000 SNAP nonlinear ultrasonic system; (**b**) Transmitting transducer; (**c**) Piezoelectric ceramics (the type: PSN-33).

In experimental measurements, two kinds of aluminum sheets with dimensions of 1.5 mm × 625 mm × 1250 mm and 2 mm × 625 mm × 1250 mm are respectively used for experimental measurements, as shown in Figure 4a. The reference trigger of the DPO 3014 digital phosphor oscilloscope (manufactured by Tektronix Inc., Beaverton, OR, USA) is triggered from the internal trigger signal of RAM-5000 SNAP (Figure 4a). A 10-cycle tone burst of 200V with “Hanning window” generated by RAM-5000 SNAP is fed into the transmitting transducer (Figure 4b), which is attached to the acrylic wedge with the angle 30° to generate *S*_0_ mode waves in aluminum sheets. The wedge is coupled to the transducer and the sheet with Glycerol. The commercial piezoelectric transducer (Model: V1012, Olympus Inc., Tokyo, Japan) with the central frequency of 250 kHz is used as the transmitter. The wave signals propagating in the sheet are received at different positions (see Figure 2) by the piezoelectric ceramics (the type: PSN-33, manufactured by Haiying Inc., Wuxi, China), as shown in Figure 4c, which are adhesively bonded to the sheet. Then the received signals are sent back to the oscilloscope through RAM-5000 SNAP. Finally, the digitized time-domain signals are saved and processed by the computer. Meanwhile, the experimental received data is filtered by both a low-pass filter (20 MHz) and a high-pass filter (50 kHz) by the RAM-5000 SNAP, and average filter (32 times) by the oscilloscope. The received signals are both amplified by a preamplifier (Model: RS-5-G2, RITEC Inc., Warwick, RI, USA) with a −20 dB gain and by a receiver amplifier with a 50 dB gain for 200 kHz and 300 kHz in a 2.0-mm sheet, or by a receiver amplifier with a 40 dB gain for 240 kHz and 300 kHz in a 2.5-mm sheet.

## 5. Result Discussion

Owing to the existing quadratic material nonlinearity, second harmonics can be generated with the propagation of the fundamental *S*_0_ mode waves. In this section, by analyzing the data from numerical simulations and experiments, the effects of different frequency-thicknesses and different frequencies with the same frequency-thickness on dispersion distance are investigated here. Note that the signals of Lamb waves in the *y*-direction (the direction perpendicular to the surface of the sheet) are used both for numerical simulations and experiments [41].

### 5.1. Fundamental Waves and Second Harmonics

**Figure 5:**
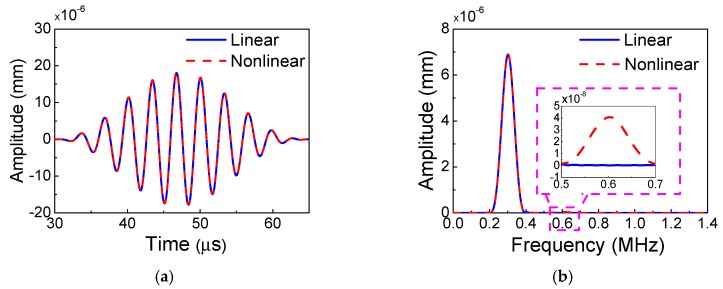
The signals received at the location of 120 mm from the numerical simulations (**a**) time domain; (**b**) frequency domain (Frequency-thickness: 600 kHz⋅mm).

**Figure 6:**
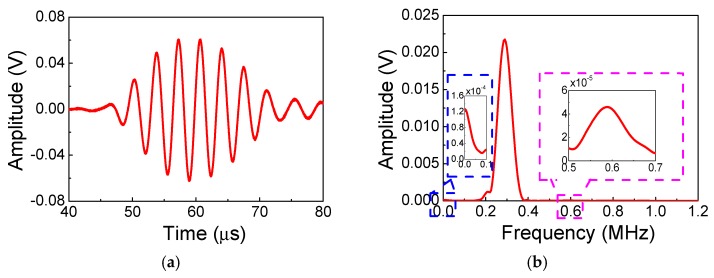
The signals received at the location 125 mm from the experiments (**a**) time domain; (**b**) frequency domain (Frequency-thickness: 600 kHz⋅mm).

Figure 5 shows the signals collected at the location of 120 mm from numerical simulations with frequency-thickness 600 kHz⋅mm (*f* = 300 kHz, *h* = 2 mm), wherein the solid line represents the wave signals for the linear case, and the dashed line represents the wave signals for the nonlinear case. Additionally, the experimental signals collected at the location of 125 mm with the same frequency-thickness are shown in Figure 6. It is found that the time-domain signals of the linear case nearly coincide with that of the nonlinear case as shown in Figure 5a. However, based on the frequency-domain signals with Fast Fourier Transform (FFT), as shown in Figure 5b, we can clearly observe that the wave signals of the nonlinear case marked by the dashed line contain both the fundamental frequency of 300 kHz and the second harmonic 600 kHz. Meanwhile, the signals of the linear case marked by the solid line only contain the fundamental frequency of 300 kHz, indicating that the second harmonic cannot be generated for the linear case. Additionally, the experimental result as shown in Figure 6 demonstrates the same phenomenon. The experimental frequency-domain signals contain not only the second harmonic but also the zero-mode frequency, as shown in Figure 6b. It is found that the amplitude of the second harmonic is rather small. However, this small second harmonic signal should not be noise since the equipment of RAM-5000 SNAP has a great advantage of being able to acquire the weak harmonics with low-noise, and the amplitude of this small second harmonic can be linearly accumulated during the certain distance. Moreover, the coincident results from repeated experiments are obtained in this work. In addition, the experimental amplitude of zero-mode frequency in the present work is smaller than that in the work of Reference [42] because of the different frequencies (300 kHz in the present work and 200 kHz in the work [42]) and different receiver transducers (piezoelectric ceramics PSN-33 in the present work and Olympus transducer with center frequency 500 kHz in the work of Reference [42]). Here, we find from the numerical simulations and experiments that, when using the low-frequency *S*_0_ mode, weak material nonlinearity is essential to the second harmonic generation of Lamb waves.

### 5.2. The Influence of Frequency-Thickness

In order to obtain repeatable experimental results and eliminate the effect due to the inhomogeneous distribution of the acoustic nonlinearity parameter in the sheet interior, multi-point and repeated measurements are performed in experiments. Each experiment shown in the following is repeated over 40 times. Besides, it should be noted that the wave signals in the distance range (0–100 mm) could not be collected due to the limitations of the experimental equipment.

**Figure 7:**
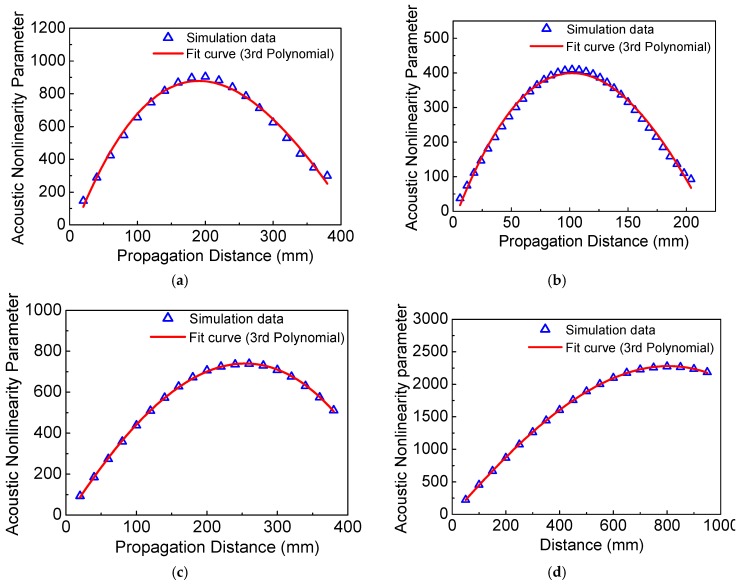
The acoustic nonlinearity parameter versus propagation distance from the numerical simulations. (**a**) *f* = 300 kHz, *h* = 2.0 mm; (**b**) *f* = 300 kHz, *h* = 2.5 mm; (**c**) *f* = 240 kHz, *h* = 2.5 mm; (**d**) *f* = 200 kHz, *h* = 2.0 mm.

**Figure 8:**
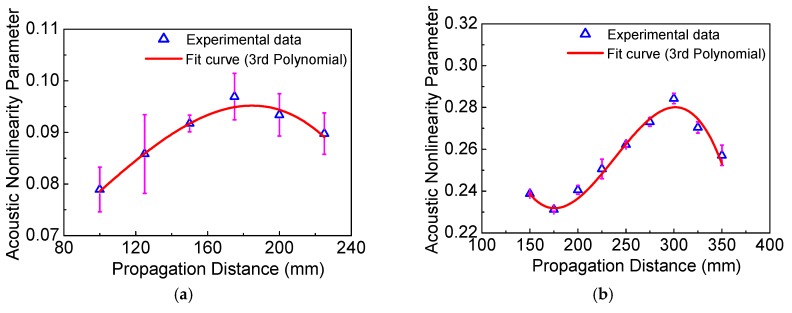

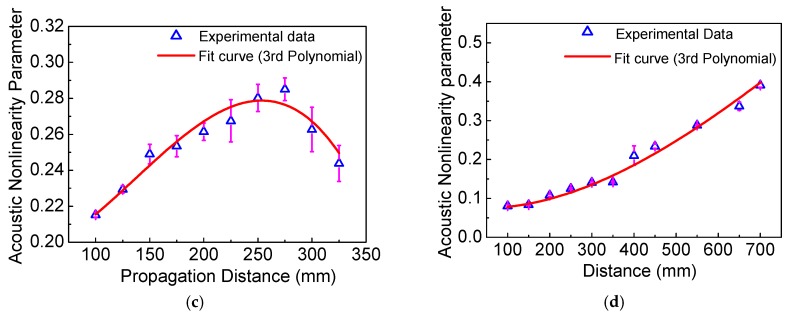
The acoustic nonlinearity parameter versus the propagation distance with standard deviations from the experiments (**a**) *f* = 300 kHz, *h* = 2.0 mm; (**b**) *f* = 300 kHz, *h* = 2.5 mm; (**c**) *f* = 240 kHz, *h* = 2.5 mm; (**d**) *f* = 200 kHz, *h* =2.0 mm.

The different curve fitting types, such as 1st, 2nd, 3rd, 4th, and 5th polynomial, are attempted to fit the numerical and experimental data in Figure 7 and Figure 8. We find that both the 1st and 2nd polynomials are inaccurate, however, the 4th and 5th polynomials show the same accuracy as the 3rd polynomial, which is expressed by a more complex style. Thus, the 3rd polynomial is the best choice of the curve fitting and is employed in Figure 7 and Figure 8 for this work.

Figure 7 and Figure 8 show the acoustic nonlinearity parameter versus the propagation distance from the numerical simulations and experiments, respectively. Note that the average values with the associated standard deviations from experiments are shown in Figure 8. It was found that the numerical and experimental results clearly show the sinusoidal behavior as in Figure 7 and Figure 8. Additionally, the dispersion distances obtained from the numerical simulations and experiments for different cases are listed in Table 2, which shows that the experimental results are consistent with those of the numerical simulations.

**Figure 9:**
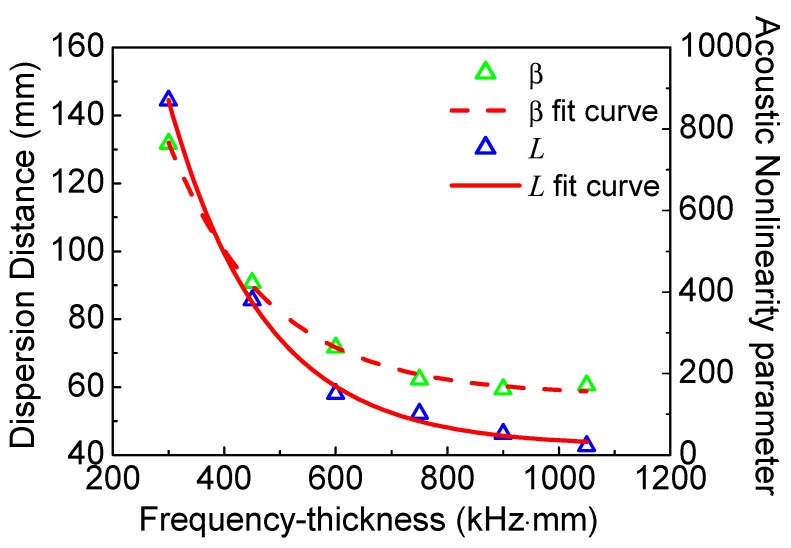
The acoustic nonlinearity parameter in the location at 10 mm and the dispersion distance versus frequency-thickness.

Meanwhile, we also find that the dispersion distance increases with the decrease of the frequency-thickness, as shown in Table 2. To investigate the relationship between dispersion distance and frequency-thickness, more numerical simulations are carried out here. The acoustic nonlinearity parameter versus frequency-thickness and dispersion distance versus frequency-thickness are shown in Figure 9. Both the acoustic nonlinearity parameter and dispersion distance decrease dramatically with the increase of the frequency-thickness, which are shown as the function of exponential decay. It is clearly shown that both the acoustic nonlinearity parameter and dispersion distance are slightly changed at relatively lower levels over 800 kHz⋅mm. Therefore, the proper frequency-thickness region should be carefully chosen from 0–800 kHz⋅mm. Note that, the lower frequency-thickness could raise the sensitivity and the linear accumulative distance, which can provide a feasible theoretical and experimental basis for nonlinear Lamb waves of the low-frequency *S*_0_ mode.

**Figure 10:**
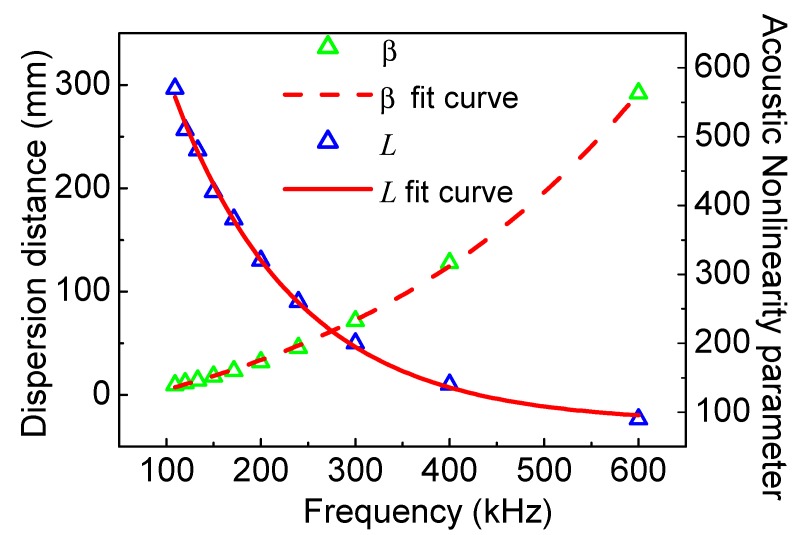
The acoustic nonlinearity parameter in the location ay 10 mm and the dispersion distance versus frequency at the same frequency-thickness 600 kHz⋅mm.

Furthermore, an interesting phenomenon can be found from Table 2, revealing the influence of frequency on dispersion distance with the same frequency-thickness. Numerical simulations are also explored to investigate the relationship. With the same frequency-thickness (600 kHz⋅mm), the acoustic nonlinearity parameter versus frequency and dispersion distance versus frequency are shown in Figure 10. The acoustic nonlinearity parameter shows an exponential increase trend with frequency; however, dispersion distance shows an exponential decay trend with frequency. We can also find that the acoustic nonlinearity parameter is more sensitive to the frequencies over 200 kHz, but dispersion distance is more sensitive to the frequencies below 400 kHz. Therefore, the appropriate frequency from the range 200–400 kHz could be a compromise considering the influences of sensitivity and linear accumulative distance.

It should be noted that the representative case (*f* = 200 kHz, *h* = 2.0 mm) has two great advantages: a lower frequency-thickness (400 kHz⋅mm) and a lower frequency (200 kHz), which can lead to a higher sensitivity and longer linear accumulative distance. The numerical and experimental results both verify the above-mentioned viewpoints.

## 6. Conclusions

The numerical modeling and experimental investigation in plates with quadratic material nonlinearity are performed to demonstrate the nonlinear phenomena of the low-frequency *S*_0_ mode Lamb wave. The following conclusions are drawn:

Firstly, when the *S*_0_ mode Lamb waves in the low-frequency domain (0–800 kHz⋅mm) are chosen as the fundamental waves, obvious second harmonics can be observed from the numerical and experimental results. Therefore, the low-frequency domain (0–800 kHz⋅mm) is an appropriate choice for engineering applications.

Secondly, it is found that both the acoustic nonlinearity parameter and dispersion distance decrease dramatically as the function of exponential decay with the increase of frequency-thickness. Thus, the lower frequency-thickness from 0–800 kHz⋅mm should be carefully chosen for considering the sensitivity and the linear accumulative distance.

Finally, in the cases of the same frequency-thickness, the appropriate frequency from the range 200-400 kHz could be a compromise considering the influences of sensibility and linear accumulative distance. Additionally, both the numerical and experimental results manifest that the frequency plays a critical role to affect acoustic nonlinearity parameter and dispersion distance. Thus, according to Figure 9 and Figure 10, the appropriate frequency should be carefully chosen in actual NDT applications. This study provides a theoretical and experimental foundation for nonlinear Lamb-wave methods based on the low-frequency *S*_0_ mode.

## Figures and Tables

**Table 1:** The mechanical parameters of aluminum AL-6061-T6.

*ρ* (kg/m^3^)	*λ* (MPa)	*μ* (MPa)	*l* (MPa)	*m* (MPa)	*n* (MPa)
2704	5.11 × 10^4^	2.63 × 10^4^	−2.82 × 10^5^	−3.39 × 10^5^	−4.16 × 10^5^

**Table 2:** The dispersion distances from numerical simulations and experiments.

Case	*L*/mm (Numerical Simulations)	*L*/mm (Experiments)	Error
*f* = 300 kHz, *h* = 2.0 mm	180	175	2.8%
*f* = 300 kHz, *h* = 2.5 mm	100	125	25%
*f* =240 kHz, *h* = 2.5 mm	240	250	4.2%
*f* = 200 kHz, *h* = 2.0 mm	800	700	12.5%
